# A novel method for semi-quantitative detection of HPV16 and HPV18 mRNA with a low-cost, open-source fluorimeter

**DOI:** 10.1007/s00216-025-05765-8

**Published:** 2025-02-07

**Authors:** Kathryn A. Kundrod, Mary E. Natoli, Chelsey A. Smith, Jackson B. Coole, Megan M. Chang, Emilie Newsham Novak, Elizabeth Chiao, Elizabeth A. Stier, Jane R. Montealegre, Michael E. Scheurer, Philip E. Castle, Kathleen M. Schmeler, Rebecca R. Richards-Kortum

**Affiliations:** 1 Department of Bioengineering, Rice University, Houston, TX USA; 2 Division of Cancer Epidemiology and Genetics, National Cancer Institute, Rockville, MD USA; 3 Department of Bioengineering, University of Washington, Seattle, WA USA; 4 Department of Epidemiology, The University of Texas MD Anderson Cancer Center, Houston, TX USA; 5 Department of Oncology, The University of Texas MD Anderson Cancer Center, Houston, TX USA; 6 Department of Obstetrics and Gynecology, Boston University Chobanian & Avedisian School of Medicine, Boston, MA USA; 7 Department of Behavioral Science, The University of Texas MD Anderson Cancer Center, Houston, TX USA; 8 Department of Pediatrics-Hematology/Oncology, Baylor College of Medicine, Houston, TX USA; 9 Division of Cancer Prevention, National Cancer Institute, Rockville, MD USA; 10 Department of Gynecologic Oncology & Reproductive Medicine, The University of Texas MD Anderson Cancer Center, Houston, Texas USA

**Keywords:** Cervical cancer, HPV testing, MRNA, Point-of-care diagnostics

## Abstract

Despite global calls to eliminate cervical cancer, rates of cervical cancer incidence and mortality remain high in resource-limited settings, where it is challenging to implement and sustain screening, diagnosis, and treatment programs. The presence of high-risk HPV mRNA in cervical cells is a sensitive and specific biomarker of cervical precancer. Yet, current testing methods are too costly and complex for use in resource-limited settings. Here, we present a novel method for semi-quantitative detection of HPV16 and HPV18 mRNA with minimal infrastructure requirements. The assay relies on isothermal reverse transcription recombinase polymerase amplification (RT-RPA) with real-time fluorescence readout, demonstrated on rugged, portable, and affordable instruments. We demonstrate adapting the assay from DNA detection to RNA detection, characterizing the test with samples of increasing biological complexity, and ultimately establishing a limit of detection of 1000 HPV16 or HPV18 transcripts per reaction with RNA extracted from cell lines. HPV16 and HPV18 mRNA assays were used to test total RNA from 11 patient samples; results for 10 samples (91%) agreed with the gold standard of RT-qPCR. To reduce cost, the assay was demonstrated with multiplexed detection of HPV16 and HPV18 DNA, validated with a reaction volume that was reduced from 50 to 5 µL with DNA and RNA, and performed using a low-cost, portable reader with DNA and RNA. With incorporation of point-of-care-friendly sample preparation and detection of additional genotypes, this test has the potential to expand global access to HPV testing.

**Supplementary Information:**

The online version contains supplementary material available at 10.1007/s00216-025-05765-8.

## Introduction

Incidence of and mortality from cervical cancer have decreased dramatically in countries with organized screening programs that enable the early detection and treatment of cervical precancer and cancer. However, an estimated 604,000 people are diagnosed with and 342,000 die from cervical cancer every year [1]. Approximately 94% of cervical cancer deaths occur in low- and middle-income countries (LMIC), where primary prevention through HPV vaccination and secondary prevention through cervical screening, diagnosis, and treatment are challenging to implement at scale [2].

In LMIC, high-risk HPV testing is the recommended screening method [3]. However, current HPV testing methods are costly and require infrastructure that is often not available in LMIC and resource-limited areas of high-income countries (HIC). Tests are available to detect HPV DNA, mRNA, or oncoproteins, and the accuracy to identify patients with cervical precancer and cancer depends on which biomarker is targeted. Tests that detect HPV DNA have high clinical sensitivity to identify patients with cervical precancer and cancer but low specificity, because they may also identify many patients with transient HPV infections [4]. In contrast, HPV oncoprotein testing is highly specific for the identification of patients with precancerous and cancerous lesions caused by persistent HPV infection. However, oncoprotein testing has limited sensitivity to identify patients with early precancers [5]. The use of mRNA testing to detect HPV E6 or E7 transcripts has the potential to achieve both high clinical sensitivity and specificity, thereby providing more accurate screening [6–10]. However, commercially available HPV mRNA tests are expensive, with per-test cost in the United States estimated to be US$74 per test (range: US$19–$290). These commercially available tests also require costly equipment (from US$45,000 to $150,000) [11, 12], depend upon advanced infrastructure (e.g., electricity, controlled climate, routine maintenance), and are qualitative. Thus, access to HPV mRNA testing is limited in LMIC and resource-constrained areas of HIC.

Here, we present a method for isothermal reverse transcription and amplification for semi-quantitative, real-time detection of HPV16 and HPV18 E7 mRNA using low-cost, rugged equipment designed for use in resource-constrained settings. To reduce the cost and complexity of mRNA detection, we employ one-pot reverse transcription recombinase polymerase amplification (RT-RPA), an isothermal amplification approach that is more tolerant of inhibitors compared to other nucleic acid amplification technologies.

We describe efforts to optimize the RT-RPA assay and assess performance with increasingly complex targets, including synthetic E7 DNA, extracted cellular DNA, *in vitro* E7 RNA transcripts, extracted cellular RNA, and extracted RNA from cervicovaginal swab samples. We present three strategies to reduce the cost of the test: (1) multiplexing HPV16 and HPV18 assays into a single reaction, (2) reducing reaction volume, and (3) performing amplification and detection using a low-cost, open-source fluorimeter that is appropriate for use in resource-constrained settings. With future incorporation of sample preparation, these advances allow for the detection of HPV16 and HPV18 mRNA using a low-cost assay with minimal infrastructure requirements—a critical step needed to support global access to cervical cancer screening with HPV mRNA testing.

## Methods

We present three studies to develop, evaluate, and reduce the cost of the real-time RT-RPA assay for the detection of HPV16 and HPV18 mRNA. First, we optimized the RT-RPA assay in exo format for real-time readout with fluorescence. Second, assay performance was evaluated and further optimized with samples of increasing biological complexity. Finally, we adapted the assay to a more point-of-care-friendly format by multiplexing targets, reducing assay volume, and demonstrating use with lower-cost instrumentation. The approach for each study is described below; details of the RPA reaction formulation for each experiment are provided in Electronic Supplementary Material Table S1.

### Optimization of real-time RT-RPA assay for detection of HPV16 and HPV18 mRNA

RPA exo kits with lyophilized enzyme pellets were obtained through TwistDx, Limited (Maidenhead, UK). For all RPA and RT-RPA exo reactions, primers and probes were prepared at 10 µM working concentrations in 1X TE. The magnesium acetate catalyst for RPA reactions was added to the cap of each reaction during assembly and was spun into the reaction mix after capping the tube using a mini centrifuge.

Primer and probe sequences are included in Electronic Supplementary Material Table S2. RPA primers and probes were based on those described previously for HPV16 and HPV18 DNA detection using the RPA nfo format with lateral flow detection [13]. RPA primers were employed here without modification; probe labels were redesigned for the RPA exo format with real-time fluorescence readout (see Electronic Supplementary Material Table S1). Primers were obtained from Integrated DNA Technologies, Inc., and probes were obtained from LGC Biosearch Technologies (Novato, CA).

To optimize the assay, all RPA reactions were run on Axxin T8-ISO and T16-ISO instruments, used interchangeably, in singleplex format. The T8-ISO and/or T16-ISO were set at 40 °C for 20 min with a 1-min read delay and 20-s sampling rate. The pulse width modulation (PWM) was set to 30%. A 2-mm grade 100 hardened AISI 420 stainless steel ball-bearing (Simply Bearings, Leigh, UK) was added to each reaction tube for continuous mixing throughout incubation, and reactions were assembled in high-profile 8-tube PCR strips provided in RPA exo kits.

To reduce false positives in the HPV16 assay, the concentration of the probe and Tte UvrD (New England Biolabs, Ipswich, MA) were optimized as described in Electronic Supplementary Material Table S1.

### Preparation of targets of increasing biological complexity

We evaluated assay performance for short synthetic DNA sequences; short *in vitro* transcribed RNA sequences; full genome DNA and RNA extracted from cell lines; and RNA extracted from cervicovaginal swabs. All targets were quantified by qPCR or RT-qPCR prior to adding to 50-µL singleplex RPA or RT-RPA reactions, as described in Electronic Supplementary Material Table S1. RPA and RT-RPA reactions were run on the T8-ISO or T16-ISO instruments, used interchangeably.

#### **Quantification of DNA and RNA targets**

The concentration of all targets was independently quantified with qPCR or RT-qPCR. All qPCR and RT-qPCR experiments were conducted on a CFX96 Touch thermocycler (Bio-Rad Laboratories, Hercules, CA). Primer sequences for the primers used in both qPCR and RT-qPCR are provided in Electronic Supplementary Material Table S2; primers were obtained from Integrated DNA Technologies, Inc. (Coralville, IA). qPCR reactions were assembled and run as previously described [13], and single-use aliquots of quantified target DNA were prepared and stored at −20 °C or −80 °C for up to 6 months, with dilutions prepared in nuclease-free water on each day of experiments.

RT-qPCR reactions were assembled with the Luna Universal One-Step RT-qPCR Kit (New England Biolabs, E3005). Each reaction contained 10 µL 2X Luna Universal One-Step Reaction Mix (2X), 1 µL Luna WarmStart RT Enzyme Mix (20X), 0.8 µL forward and 0.8 µL reverse primer (each at a 10 µM working concentration in 1X TE), 2.4 µL nuclease-free water, and 5 µL purified sample. The thermocycling protocol included the following steps: 55 °C for 10 min, 95 °C for 1 min, 40 cycles of 95 °C for 10 s, and 60 °C for 1 min with a plate read during each cycle, followed by a melt curve.

HPV16 and HPV18 E7 RNA concentrations from both *in vitro* transcripts and extracted cellular RNA were estimated with RT-qPCR against DNA standards. Reverse transcription efficiency was assumed to be 100%. Following quantification, single-use RNA aliquots were prepared in nuclease-free water and stored at −80 °C, and dilutions were prepared in nuclease-free water on the same day as use.

#### **Short, synthetic DNA sequences**

Single-use aliquots of synthetic gBlock DNA were prepared and quantified as previously described [13]. Briefly, gBlocks Gene Fragment (gBlock) DNA for the full E7 genes of HPV 6, 11, 16, 18, 31, 33, 35, 39, 45, 51, 52, 56, 58, 59, 68, 72, and 82 based on the sequences published in the PapillomaVirus Episteme (PaVE; available pave.niaid.nih.gov) were purchased from Integrated DNA Technologies Inc. (Coralville, IA). HPV16 and HPV18 gBlocks were quantified by qPCR against quantitative synthetic DNA standards acquired from the American Type Culture Collection (ATCC, VR-3240SD, and VR-3241SD). gBlocks for remaining HPV genotypes were quantified via NanoDrop ND-1000.

#### **Full genome DNA extracted from cell lines**

SiHa and HeLa cells were acquired from ATCC (HTB-35 and CCL-2, respectively, Manassas, VA). Cells were passaged up to ten times, pelleted in quantities of 0.5–10 million cells, and stored at −80 °C until use. DNA extracted from SiHa and HeLa cell lines was prepared and quantified as previously described [13]. Briefly, DNA was extracted from cells using a DNeasy Blood and Tissue kit (Qiagen) per the manufacturer’s instructions, including final elution into 200 µL of nuclease-free water, and quantified against ATCC quantitative DNA standards (VR-3240SD and VR-3241SD) via qPCR.

#### **In vitro transcribed RNA sequences**

To transcribe RNA, gBlocks were designed for HPV16 and HPV18 E7 regions along with T7 promoter sequences and were purchased from IDT. E7 sequences were identified through the PapillomaVirus Episteme (PaVE, available: pave.niaid.nih.gov) [14, 15]. gBlocks were transcribed *in vitro* via the TranscriptAid T7 High Yield Transcription Kit (Thermo Fisher Scientific K0441) per manufacturer’s instructions, including digestion by DNase I for 30 min at 37 °C. Following DNase digestion, RNA was purified with the Monarch RNA Cleanup Kit (New England Biolabs, Inc. T2040) per manufacturer’s instructions with a final elution step into 100 µL of nuclease-free water.

#### **Full genome RNA extracted from cell lines**

Total RNA was extracted from SiHa and HeLa cells using the Monarch Total RNA Miniprep Kit (New England Biolabs, Inc., T2010) per manufacturer’s instructions for cultured mammalian cells with final elution into 100 µL of nuclease-free water. Eluted RNA was combined with RNase-free DNase I (Thermo Fisher Scientific, EN0521) at a DNase I concentration of 0.1 U/µL, and the reaction was incubated at 37 °C for 30 min. Following DNase I digestion, RNA was purified with the Monarch RNA Cleanup Kit (New England Biolabs, Inc. T2040) per manufacturer’s instructions with a final elution step into 100 µL of nuclease-free water.

#### **RNA extracted from cellular samples collected with cervicovaginal swabs**

Cellular samples were collected with cervicovaginal swabs into PreservCyt media (Hologic, Marlborough, MA) from participants in a multi-center AIDS Malignancy Consortium trial focused on screening HIV-infected women for anal cancer precursors [16]. De-identified samples from the trial were transferred from the AIDS Malignancy Consortium (AMC) to Rice University. Upon receipt, samples were stored at −80 °C. Samples were then processed for DNA genotyping using the Genotype 15 High Risk HPV by Fluorescent Detection kit (Atila BioSystems GHPVF-100, Mountain View, CA). Two hundred microliters of primary samples in PreservCyt media was aliquoted and spun for 10 min at 12,500 RPM to pellet cells. Supernatant was removed without disturbing the cell pellet. Pellets were reconstituted in 20 µL of lysis buffer provided in the Atila Biosystems kit. Samples were then processed and tested per manufacturer instructions using a Bio-Rad CFX96 thermocycler.

Following genotyping, all HPV16- and HPV18-positive samples and a random subset of samples that tested negative for HPV or positive for other genotypes were tested in RT-qPCR to quantify HPV16 and HPV18 RNA. One hundred microliters of the primary PreservCyt sample was aliquoted and pelleted by centrifuging cells for 5 min at 5000 g. Supernatant was removed, and total RNA was extracted as described in the “*Full genome RNA extracted from cell lines*” section*.*

Extracted RNA was amplified in the HPV16, HPV18, and beta-actin RT-qPCR assays described in the “*Quantification of DNA and RNA targets*” section and with primers included in Electronic Supplementary Material Table S2 to determine HPV16 and/or HPV18 positivity and sample adequacy. Finally, extracted RNA was amplified in 50 µL singleplex HPV16 and HPV18 RT-RPA reactions on the T8-ISO instrument.

#### **Statement of human and animal rights**

Because samples provided by the AMC were pre-existing and de-identified, the research was determined not to be human subjects research by the Rice University Institutional Review Board.

### Reducing assay cost

Three approaches were pursued to reduce the cost of real-time RPA reactions: (1) combining the HPV16 and HPV18 assays into a single, multiplexed 50 µL reaction, (2) reducing the singleplex reaction volume, and (3) translating the assay to a low-cost benchtop heater and open-source fluorimeter.

#### **Multiplexing 50 µL reactions**

Primer and probe concentrations were varied in the multiplexed assay to minimize spurious product formation and maximize signal from target DNA (see Electronic Supplementary Material Table S1, Section 1).

#### **Reducing total reaction volume**

Smaller volume reactions were assembled by preparing a master mix for a discrete number of reactions, adding the same discrete number of enzyme pellets to the master mix, and mixing well. To prepare small-volume reactions, the master mix was added to each tube as 75% of the reaction volume, the target was added to each tube as 20% of the total reaction volume, and 280 mM magnesium acetate was added to the lid of each reaction as 5% of the total reaction volume (see Electronic Supplementary Material Table S1, Section 3).

#### **Demonstrating assay performance with lower-cost, open-source fluorimeters**

The assay was initially implemented on three commercially available instruments: a traditional thermocycler (Bio-Rad CFX96) and two commercially available isothermal fluorimeters that allowed for reaction mixing (Axxin T8-ISO and T16-ISO). To further reduce cost, assay performance with small-volume reactions was performed with a low-cost, open-source fluorimeter that costs approximately US $450 to assemble and is used with a benchtop heater [17]. Results were compared to those obtained with the Bio-Rad CFX96 Touch. Small-volume reactions were not pursued using the T8-ISO and T16-ISO instruments because the optical module on both instruments requires a minimum reaction volume of 30–50 µL [18]

On both the Bio-Rad CFX96 Touch and the open-source fluorometer, reactions were initiated by a quick-spin and vortex step, and no further agitation was performed throughout the duration of the incubation period. The Bio-Rad CFX96 was set to 40 °C for 20 min with a heated lid set at 42 °C. The heat block used with the low-cost, open-source isothermal fluorimeter was set at 40 °C for 20 min.

## Results

First, we describe results to optimize the isothermal amplification assay; second, we summarize performance for real-time detection of HPV16 and HPV18 E7 DNA and RNA with samples of increasing complexity; finally, we demonstrate assay performance in lower-cost formats.

### Optimized assay for real-time RT-RPA assay for detection of HPV16 and HPV18 mRNA

Previously, we demonstrated HPV16 and HPV18 primers and probes for an RPA nfo assay [13], which is compatible with lateral flow detection. Here, we adapted that assay to the RPA exo format, which is compatible with real-time fluorescence detection, and described the steps required to optimize the assay, first for DNA and then for RNA targets. With manufacturer-recommended conditions, the HPV16 exo assay initially demonstrated spurious primer-probe interactions when performed using the T8-ISO or T16-ISO, leading to false positives. To suppress false positives while maintaining a low limit of detection, we (1) added Tte UvrD helicase to the reaction (see Electronic Supplementary Material Fig. S1**-A**) and (2) reduced the amount of probe per reaction (see Electronic Supplementary Material Fig. S1**-B**). Tte UvrD helicase is an enzyme that unwinds double-stranded DNA in isothermal amplification reactions and can improve assay specificity [19]. The HPV18 assay sensitively detected synthetic DNA without producing false positive results on the T8-ISO and T16-ISO with manufacturer-recommended conditions. Therefore, we did not optimize the HPV18 assay further.

### Analytical sensitivity and specificity of the RPA exo assay with targets of increasing biological complexity

The developed assay was optimized using targets of increasing biological complexity. First, in the simplest configuration, the assay was characterized with short, synthetic DNA targets. Step-wise, the assay was evaluated and re-optimized as needed with full genome DNA; short, synthetic RNA; full genome RNA extracted from cultured cells; and finally, full genome RNA extracted from cervicovaginal swab samples.

#### **Short, synthetic DNA**

**Fig. 1:**
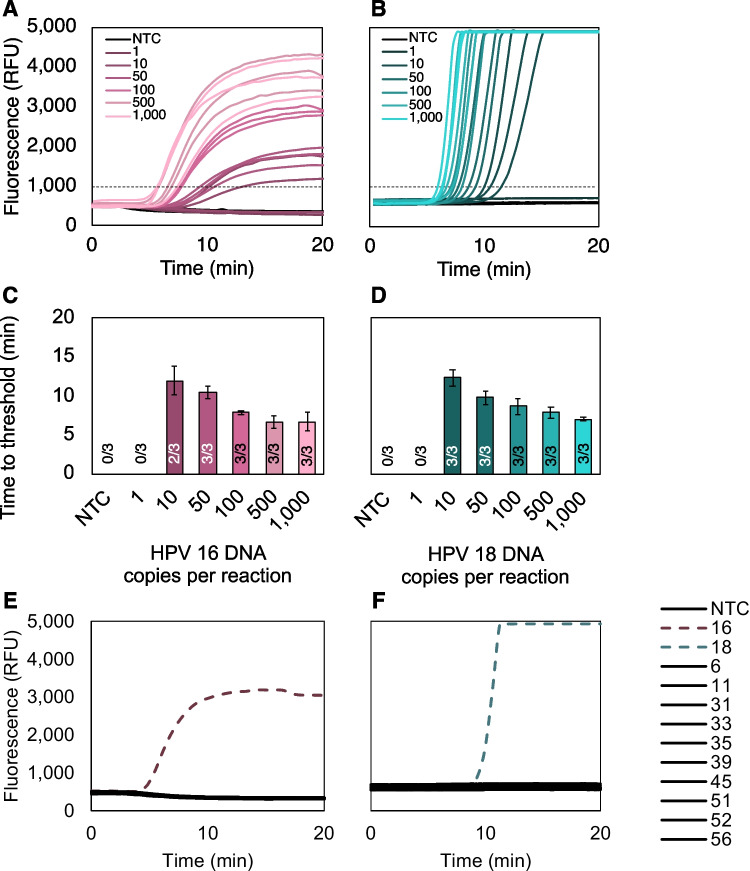
Analytical sensitivity and specificity with synthetic DNA in singleplex 50 µL reactions. The limits of detection were determined to be **A** 50 copies of HPV16 DNA per reaction and **B** 10 copies of HPV18 DNA per reaction. All copy numbers were tested in triplicate. **C–D** Time to threshold for each assay. All targets that amplified reached the positivity threshold between 6.5 and 12.5 min after fluorescence measurements were initiated on the Axxin T8-ISO or T16-ISO. A number of replicates that amplified out of the total replicates tested are shown for each copy number. Mean ± standard deviation; *n*=3 for each copy number. **E–F** Only the targeted genotype amplified among a panel of other high- and low-risk genotypes. NTC, no-target control; RFU, relative fluorescence units

The limit of detection and time to threshold were evaluated for the optimized HPV16 and HPV18 assays on the T8-ISO and T16-ISO instruments. The HPV16 assay showed a limit of detection of 50 copies per reaction with a 298-base pair target for the E7 gene (Fig. 1A). The HPV18 assay showed a slightly more sensitive limit of detection of 10 copies per reaction with a 328-base pair target for the E7 gene (Fig. 1B). Over the copy number range of 10–1000 copies per reaction, all targets that amplified reached the positivity threshold between 6.5 and 12.5 min from the start of the read window (Fig. 1C–D). The positivity threshold was set at 1000 relative fluorescence units (RFU) for both assays. We then evaluated the analytical specificity of each assay by testing performance with synthetic E7 DNA from a panel of 14 different high- and low-risk HPV genotypes. Only DNA corresponding to the targeted genotype was amplified in each assay, demonstrating high analytical specificity (Fig. 1E–F).

#### **Full genomic DNA extracted from cell lines**

**Fig. 2:**
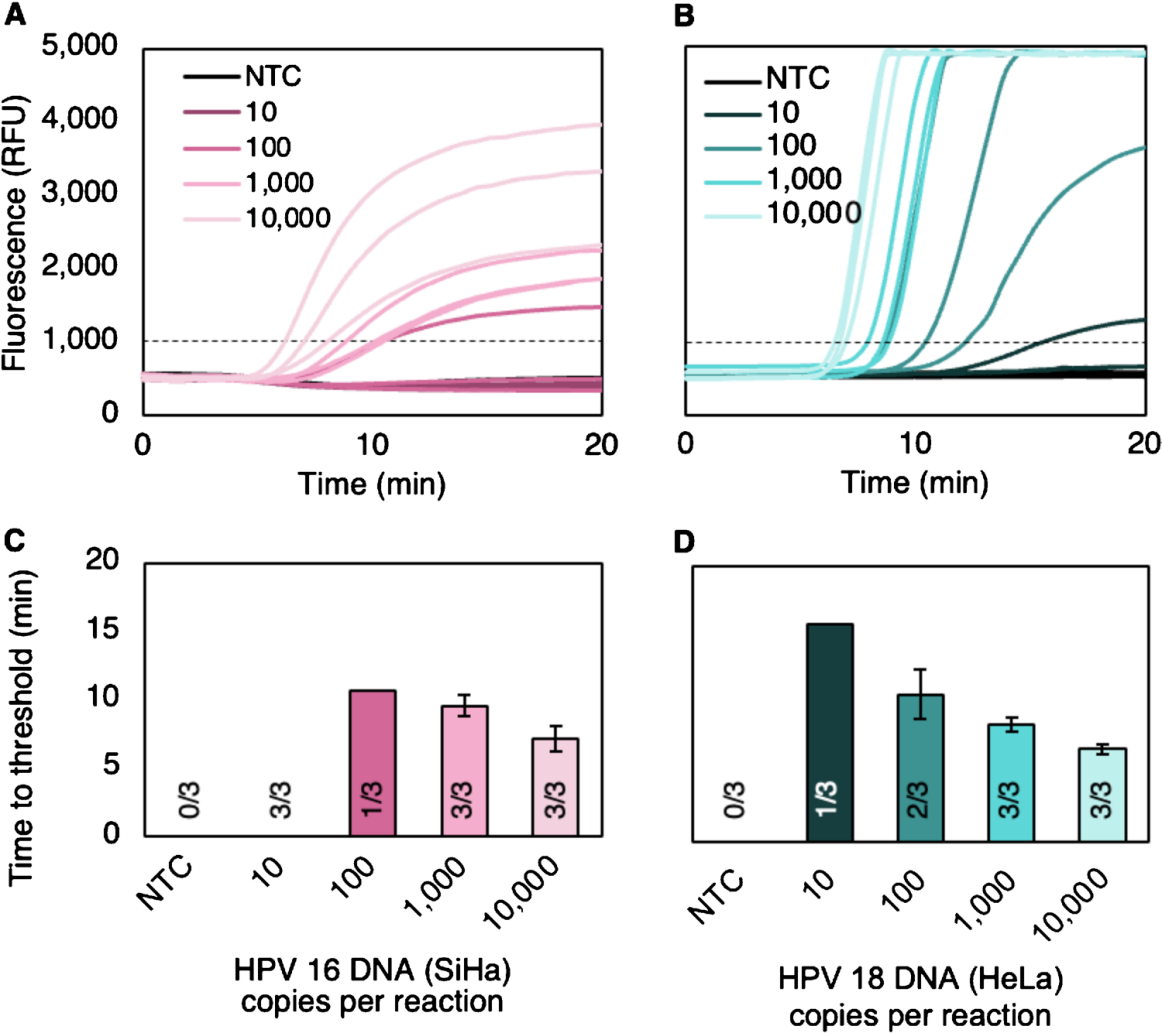
Analytical sensitivity with DNA extracted from cell lines in singleplex 50 µL reactions. The limits of detection were determined to be **A** 1000 copies of HPV16 DNA extracted from SiHa cells per reaction and **B** 100 copies of HPV18 DNA extracted from HeLa cells per reaction. **C–D** All targets that amplified reached the positivity threshold between 7 and 15 min after fluorescence measurements were initiated on the Axxin T8-ISO or T16-ISO. A number of replicates that amplified out of the total replicates tested are shown for each copy number. Mean ± standard deviation; *n*=3 for each copy number. NTC, no-target control; RFU, relative fluorescence units

Cellular DNA was extracted from SiHa and HeLa cells, which contain integrated copies of HPV16 and HPV18, respectively, in their genomes. With the longer DNA targets extracted from cell lines, the HPV16 and HPV18 RPA exo assays showed a higher limit of detection of 1000 HPV DNA copies per reaction (Fig. 2A–B). Time to threshold remained consistent between 6.5 and 12.5 min from the start of the read window (Fig. 2C–D). Semi-quantitative detection was observed for both synthetic and cellular DNA targets, with increased target numbers resulting in decreased time-to-threshold.

#### **Short, synthetic RNA**

**Fig. 3:**
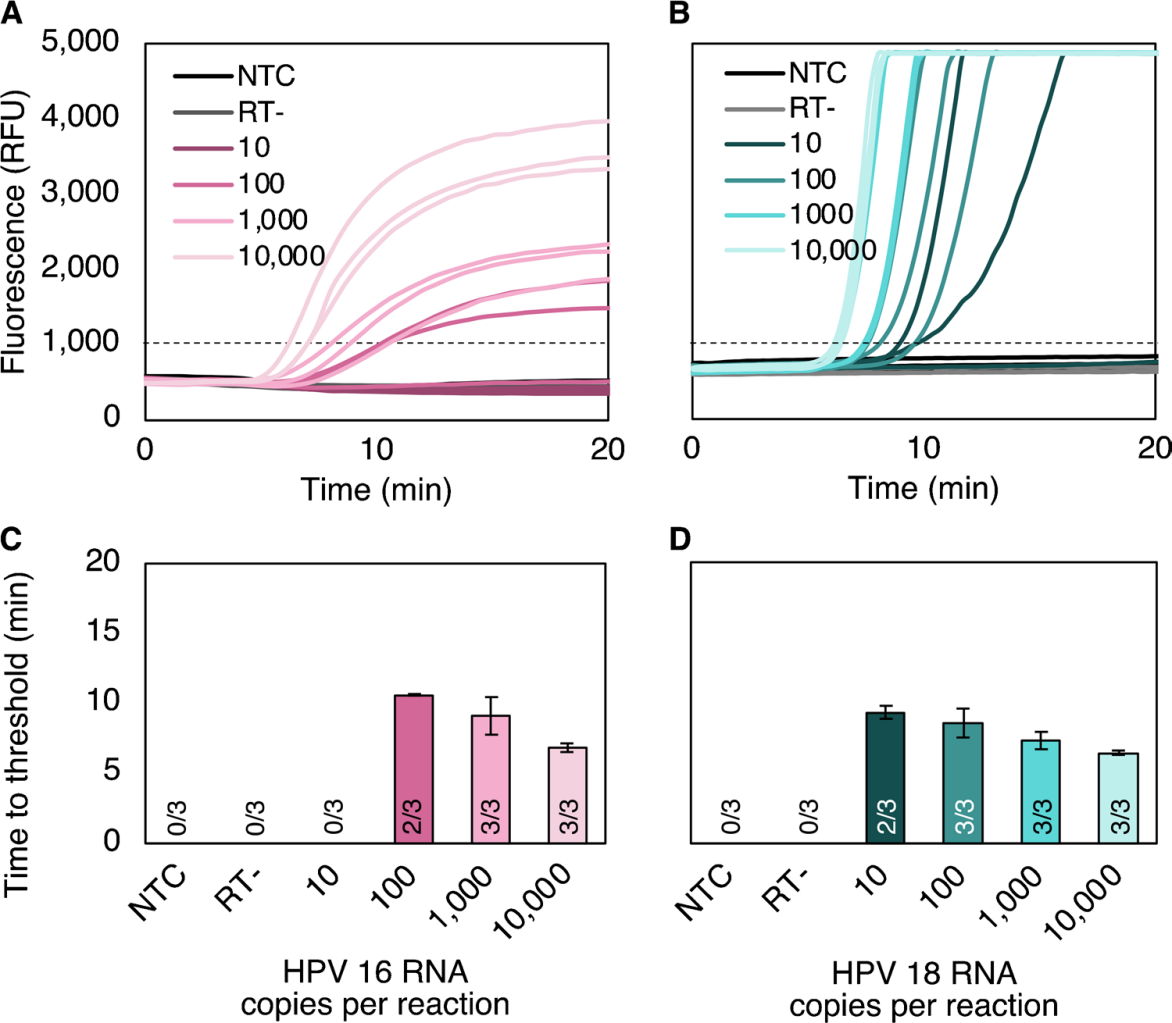
Limit of detection with *in vitro* transcribed RNA. **A** The HPV16 assay consistently detects 1000 *in vitro* transcripts per reaction, with inconsistent detection at 100 transcripts per reaction. **B** The HPV18 assay consistently detects 100 *in vitro* transcripts per reaction with inconsistent detection at 10 transcripts per reaction. No-reverse-transcriptase (RT-) controls included 10,000 transcripts per reaction and did not amplify, confirming successful DNase treatment of RNA transcripts within the copy number range tested. **C–D** Time to threshold remained consistent with DNA detection over the same copy number range between 6.5 and 12.5 after fluorescence measurement was initiated. Mean ± standard deviation of replicates that amplified, specified by the number within each bar. NTC, no-target control; RFU, relative fluorescence units; RT-, no-reverse-transcriptase control

To detect RNA targets instead of DNA, a reverse transcriptase was added directly into the RPA exo master mix. In addition, based on previously reported methods for one-step RT-RPA [20], RNaseH was added. RNaseH hydrolyzes the phosphodiester bonds of RNA when it is bound to DNA during reverse transcription, freeing up reverse-transcribed DNA for primer binding and subsequent amplification. Finally, we optimized dNTP concentration (see Electronic Supplementary Material Fig. S2), finding improved assay sensitivity with the addition of 3 mM dNTPs. We first evaluated the assay limit of detection with *in vitro* transcripts, with target lengths of 318 bases for HPV16 and 338 bases for HPV18. With *in vitro* transcripts, we found consistent detection at 1000 HPV16 RNA copies and 100 HPV18 RNA copies per reaction. We observed inconsistent detection (2/3 replicates) at 100 HPV16 RNA copies and 10 HPV18 RNA copies per reaction (Fig 3A–B). RNA detection was semi-quantitative with a comparable time to amplification as DNA ranging from 6.5 to 12.5 min (Fig. 3C–D).

#### **Full genome RNA extracted from cell lines**

**Fig. 4:**
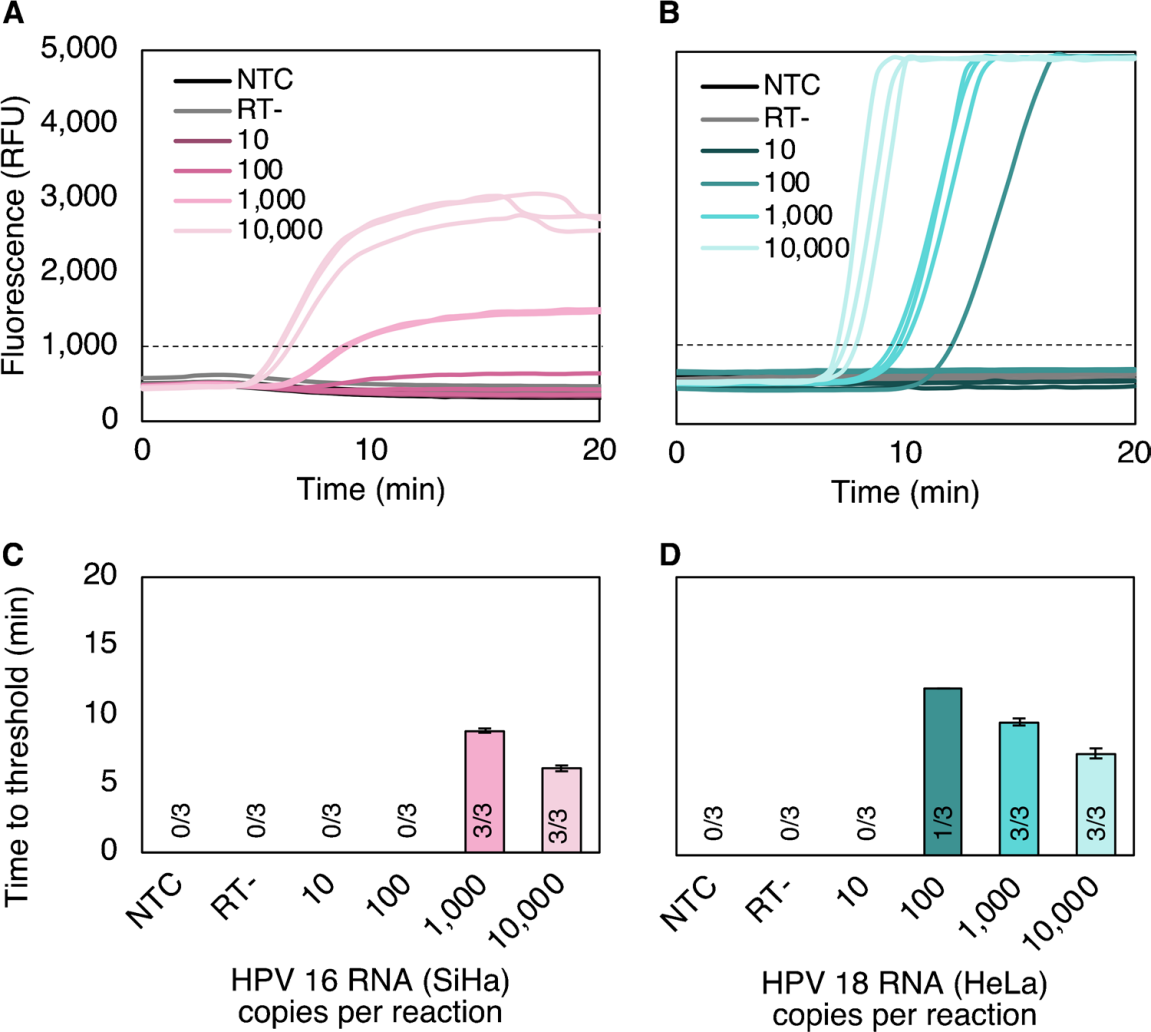
Limit of detection with RNA extracted from cell lines. **A** Per reaction, the HPV16 assay consistently detects 1,000 E7 RNA copies extracted from SiHa cells. **B** The HPV18 assay consistently detects 1000 E7 RNA copies extracted from HeLa cells, with inconsistent detection at 100 copies per reaction. All no-reverse-transcriptase controls contained 10,000 copies of E7 RNA per reaction and did not amplify, confirming successful DNase treatment of RNA targets within the copy number range tested. **C–D** Time to threshold ranged between 6.5 and 14 after fluorescence measurement was initiated. Mean ± standard deviation; *n*=3 for each copy number. NTC, no-target control; RFU, relative fluorescence units; RT-, no-reverse-transcriptase control

The limit of detection with RNA extracted from SiHa and HeLa cells was then assessed. With the longer cellular RNA targets, consistent detection was observed at 1000 HPV16 and HPV18 copies per reaction (Fig. 4A–B). Detection was semi-quantitative, and time to threshold ranged from 6 to 14 min (Fig. 4C–D).

#### **RNA extracted from patient cervicovaginal samples**

**Fig. 5:**
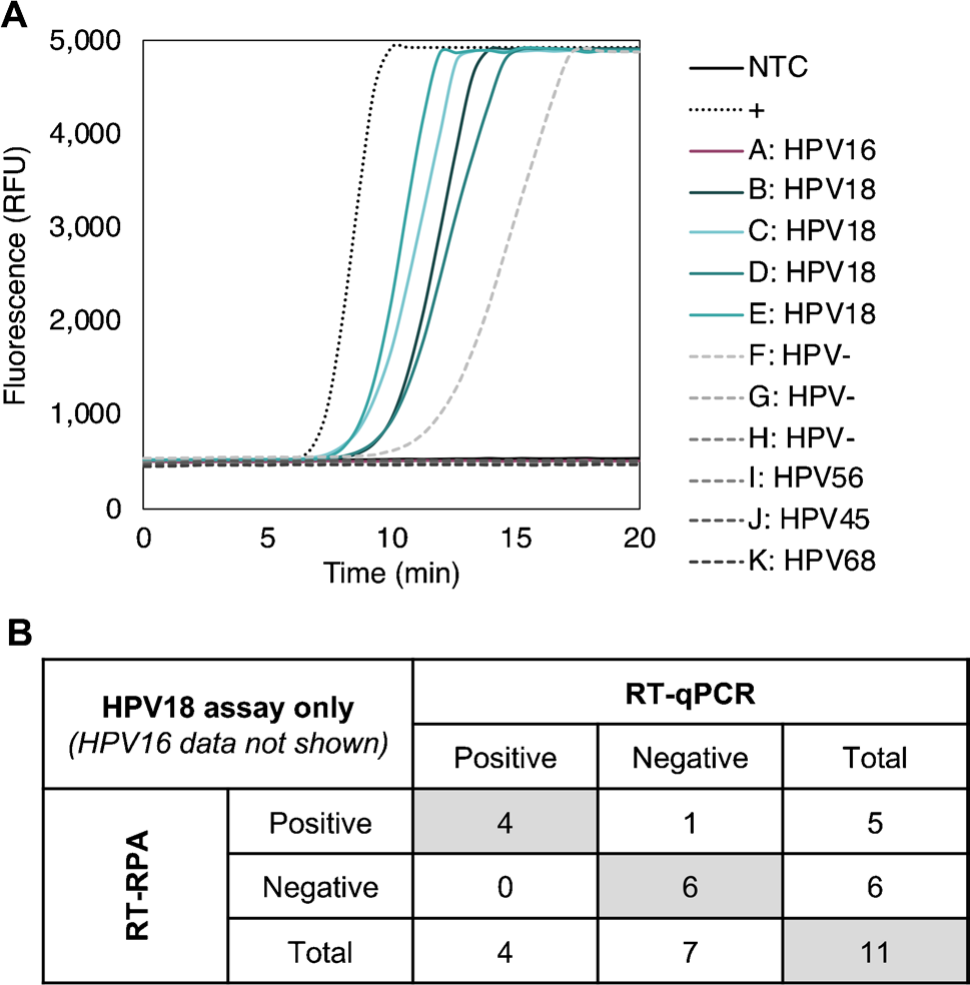
Pilot clinical evaluation of the HPV18 assay. **A** Baseline-subtracted RT-RPA curves of RNA extracted from patient cervicovaginal swabs for samples that contained HPV16, HPV18, other high-risk HPV genotypes, and high-risk HPV-negative samples, along with a positive (+) and no-target control (NTC), tested in the HPV18 assay. **B** HPV18 RT-RPA results were concordant with RT-qPCR positivity in four of five HPV18-positive samples and seven of seven HPV18-negative samples. One false positive was detected in a high-risk HPV-negative sample

Eleven cervical cellular samples collected with cervicovaginal swabs were HPV genotyped via the Atila AmpFire genotyping assay. RNA from those samples was quantified with RT-qPCR. One sample tested positive for HPV16 RNA, four tested positive for HPV18 RNA, and the remainder tested negative for HPV16 and HPV18 RNA. The five positive samples, along with three HPV-negative samples and three samples that were positive for other high-risk HPV genotypes, were tested in the developed RT-RPA exo assays for HPV16 and HPV18 using 50 µL singleplex reactions on the Axxin T8-ISO instrument. In the HPV16 assay, the single HPV16 sample and the 10 HPV16-negative samples all tested negative (data not shown). Data for the HPV18 assay are shown in Fig. 5; the four HPV18 RNA-containing samples tested positive, and all other samples tested negative.

### Assay performance at reduced cost

To reduce assay cost, we optimized the assay for multiplexed detection of both HPV16 and HPV18 in a single reaction, explored small-volume reactions that minimize the cost of required enzymes and primers, and performed the reaction using a low-cost, open-source isothermal fluorimeter.

#### **Multiplexing 50 µL reactions**

**Fig. 6:**
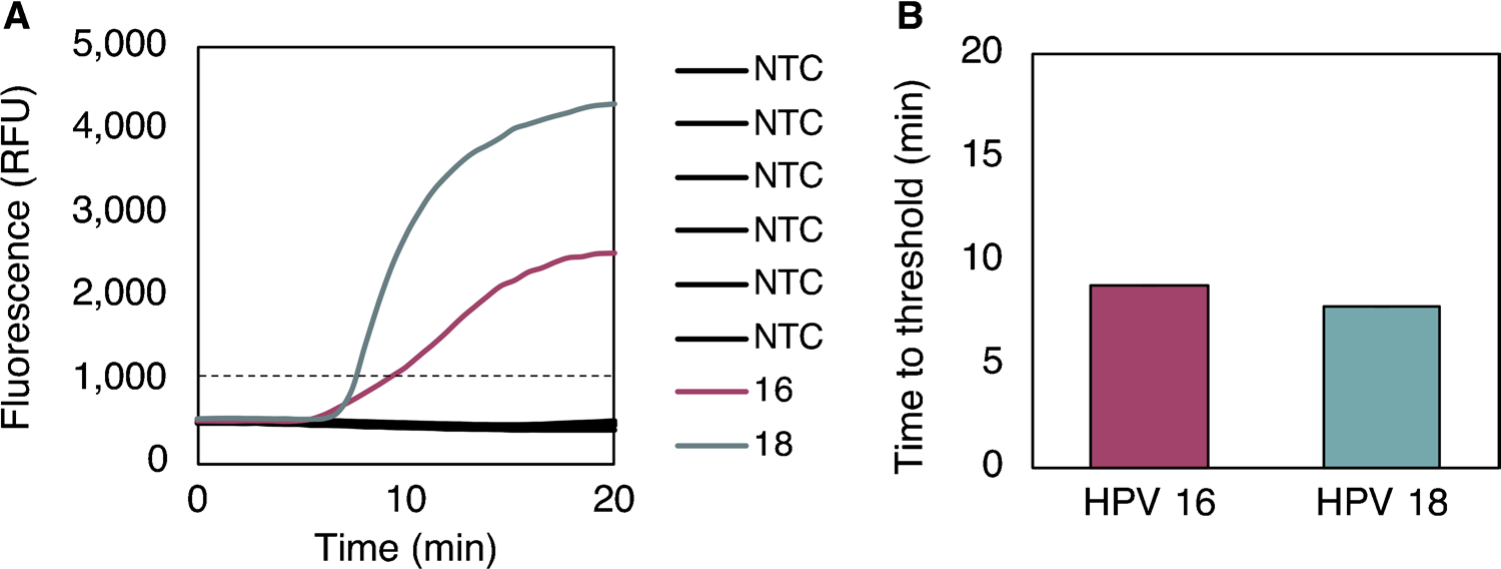
Performance of optimized multiplex assay with synthetic DNA.** A** Fluorescence curves for six no-target controls (NTCs), one HPV16 DNA positive control, and one HPV18 DNA positive control—both at 10,000 copies/reaction. NTCs remain flat over the 20 min of incubation, indicating successful false-positive suppression. **B** HPV16- and HPV18-positive controls (*n*=1 for each target) both amplify within 10 min

We first optimized primer and probe concentrations within multiplexed reactions to suppress nonspecific amplification and minimize time to threshold for both HPV16 and HPV18 DNA (see Electronic Supplementary Material Fig. S3). The optimal conditions included 158 nM forward and reverse primers for both HPV16 and HPV18, 0.3 nM HPV16 probe, and 0.6 nM HPV18 probe. The performance of the optimized multiplexed assay with synthetic DNA is shown in Fig. 6, with amplification for 10,000 copies/reaction of HPV16 or HPV18 DNA occurring within 10 min and with 0 of 6 no-target controls amplifying.

#### **Reducing total reaction volume**

**Fig. 7:**
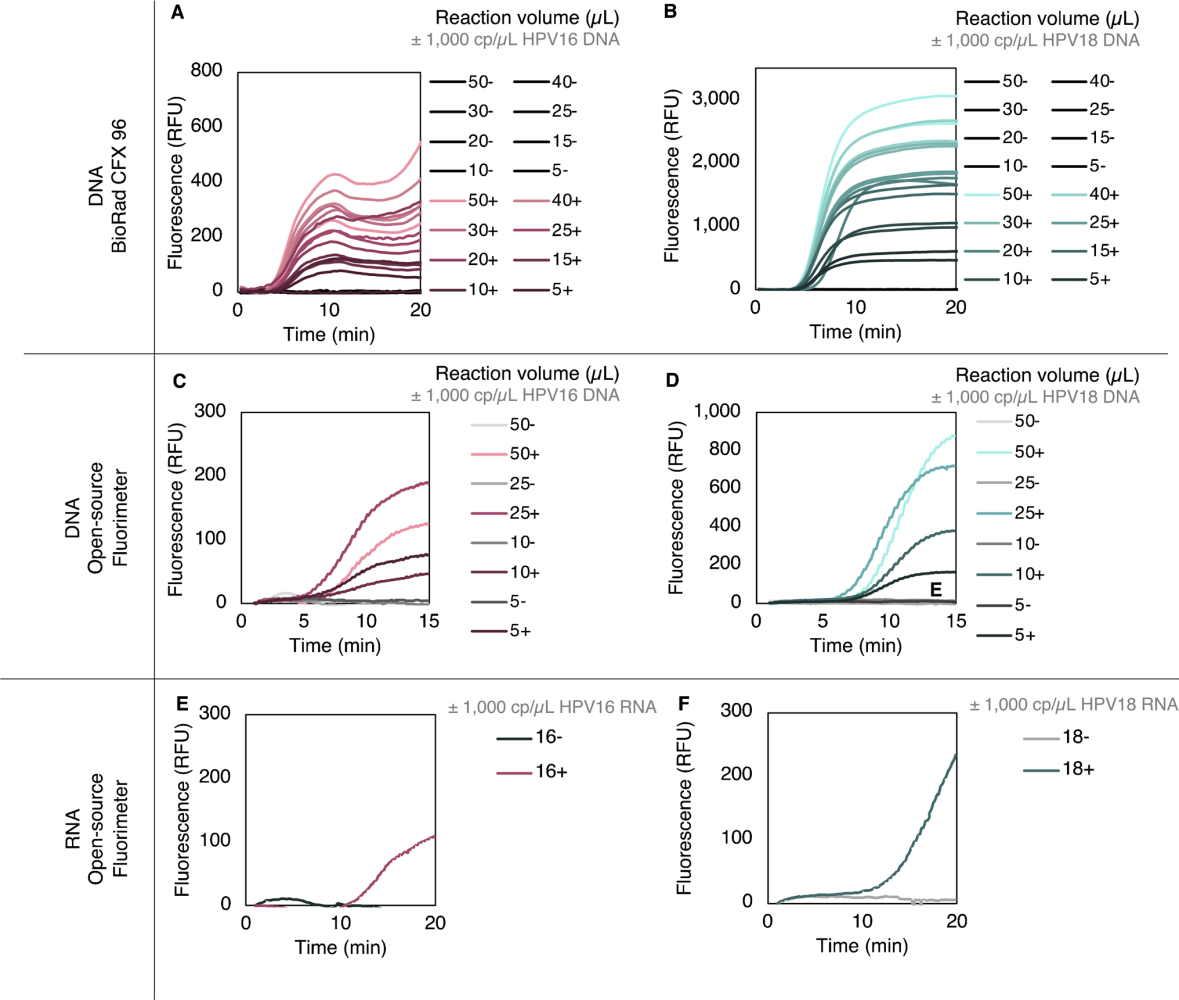
Performance of assay with small volume reactions and synthetic DNA and RNA.** A–B** Range of reaction volumes incubated and measured on the Bio-Rad CFX96 Touch. Two positive synthetic DNA samples and two no-target controls (NTCs) were included for each reaction volume; all negatives remained negative, and all positives—except for one out of two 5 µL HPV16 reactions—tested positive with a threshold at 50 RFU. The minimum reaction volume was determined to be 10 µL for HPV16 and 5 µL for HPV18. **C–D** Range of reaction volumes incubated and measured on the low-cost, open-source fluorimeter. All positive samples for reaction volumes between 5 and 50 µL are differentiable from negative samples for **C** HPV16 and **D** HPV18. **E–F** Detection of 5000 **E** HPV16 or **F** HPV18 *in vitro* transcripts per singleplex reaction on the low-cost, open-source fluorimeter. All data are baseline-subtracted

Next, we explored reductions in reaction volume, as smaller volume reactions could substantially reduce per-test cost (Table 1**,** see Electronic Supplementary Material Table S3). Singleplex reactions ranging from 5 to 50 µL in total reaction volume were tested on the Bio-Rad CFX96 Touch (Fig. 7A–B) and open-source fluorimeter (Fig. 7C–D) with DNA targets to determine the minimum reaction volume at which fluorescence could reliably be detected. On both instruments, the minimum reaction volume was determined to be 5 µL, with the exception of the HPV16 assay on the Bio-Rad CFX96 Touch, which had a minimum reaction volume of 10 µL. Additional experiments are required to validate the reduced reaction volume range with RNA.

**Table 1:** Assay cost as a function of minimum demonstrated reaction volume on each fluorimeter

**Instrument**	**Reaction volume (µL)**	**Singleplex or multiplex**	**Total reagent cost for HPV16 and 18 test (USD)**^**a**^	**Instrument cost (USD)**
T8-ISO or T16-ISO	50	Multiplex	$8.89	$6,500-8,500^b^
CFX96 Touch	10	Singleplex	$3.47	$50,000^b^
Low-cost, open-source fluorimeter	5	Singleplex	$1.74	$450^c^

^a^Cost estimate breakdowns included in Table S3

^b^List price, 2021

^c^Estimated materials cost

#### **Small-volume RNA assay demonstration on low-cost, open-source instrumentation**

Singleplex 25 µL reactions containing 1000 HPV16 or 18 *in vitro* RNA transcripts per µL were detected on the low-cost, open-source fluorimeter (Fig. 7E-F).

## Discussion

We developed a semi-quantitative isothermal reverse transcription and amplification strategy with real-time detection of HPV16 E7 and HPV18 E7 DNA and RNA. We demonstrated that the assay has high analytical sensitivity and specificity using synthetic DNA targets. We then characterized the change in analytical sensitivity with increasingly complex targets: extracted cellular DNA, *in vitro* transcribed RNA, and extracted cellular RNA. In a small pilot study, we demonstrate the ability to match RT-qPCR results for the detection of HPV18 RNA in RNA extracted from patient cervicovaginal swabs. Finally, we demonstrated strategies to reduce assay costs, including multiplexing the HPV16 and HPV18 assays into a single reaction, reducing the reaction volume, and implementing the assay with low-cost, open-source equipment designed to be used at the point-of-care.

For both HPV16 and HPV18 assays, we demonstrate the ability to detect 100–1000 *in vitro* RNA transcripts and 1000 E7 transcripts within extracted total RNA from HPV-positive cell lines. Analytic sensitivities for all target types are included in Electronic Supplementary Material Table S4. Based on the reported analytical and clinical sensitivity of commercially available HPV mRNA tests (see Electronic Supplementary Material Table S5), the limit of detection of our assay with HPV mRNA (1000 HPV16 or HPV18 transcripts per reaction) is appropriate for cervical cancer screening applications and is comparable to commercially available tests. In a systematic review of commercially available HPV mRNA tests [21], clinical sensitivity for precancer and cancer (CIN2+) detection was found to be higher for the Aptima test compared with PreTect HPV-Proofer or QuantiVirus, while PreTect HPV-Proofer showed higher clinical specificity compared with Aptima and QuantiVirus, likely due to the PreTect HPV-Proofer detecting fewer HPV genotypes [21]. Despite differences in clinical sensitivity and specificity, all three tests adequately detect cervical precancer and cancer. Considering the range of limits of detection among the commercially available tests, the limit of detection for the assay presented here corresponds to a clinically relevant number of HPV16 and HPV18 E7 mRNA transcripts. In our pilot study with participant-derived samples, we showed the detection of HPV18 RNA in cervicovaginal swab samples collected into PreservCyt media. An expanded clinical study is required to confirm acceptable clinical sensitivity and specificity, especially for HPV16 RNA.

While HPV16 and HPV18 account for 70% of invasive cervical cancers, the assay must be expanded to include additional genotypes for clinical utility. Aligned with the WHO target product profile (TPP) for HPV tests released in 2024 [22], we anticipate the need to incorporate a minimum of six additional high-risk HPV genotypes along with a cellular control for sample adequacy determination. This can likely be accomplished with a test format including three to four individual reactions with two to three multiplexed assays in each reaction.

Finally, additional work is needed to optimize the test format to minimize cost and complexity. The cost of the assay is driven primarily by two factors: reaction volume and degree of multiplexing. Effects of these two factors on reagent cost are demonstrated in Electronic Supplementary Material Table S3. With two singleplex 50 µL reactions per test—one for HPV16 and one for HPV18—the total reagent cost would be approximately US$17 per test. In this report, we demonstrated proof-of-concept detection of HPV16 and HPV18 RNA in singleplex 25 µL reactions on the open-source fluorimeter and of HPV16 and HPV18 DNA in multiplex 50 µL reactions on the T8/T16—both of which reduce reagent cost. All cost estimates are based on off-the-shelf costs for small-scale orders, and greater cost reduction is likely possible at scale. With the potential for substantial cost reduction through multiplexing and reduced reaction volume, it is of high priority to further optimize small-volume, multiplexed assay formats.

In translating our assay to smaller volumes on a low-cost (~US $450 for materials) open-source fluorimeter [17], we demonstrated amplification without any agitation during the incubation period, simplifying the workflow relative to the manufacturer-recommendation of vortexing reactions 4 min into incubation. Without agitation during incubation, we maintained the ability to detect both DNA and RNA targets. These results are consistent with previous work demonstrating that reducing RPA reaction volume can circumvent the need for agitation during the reaction [23].

For further optimization, this assay may benefit from adapting to enzymatic recombinase amplification (ERA). The ERA technology leverages the same core principles as RPA with optimized enzymatic structures and functions for increased sensitivity and reduced reaction time. A recent report demonstrated highly sensitive HPV16 and HPV18 DNA detection in real-time and lateral flow formats [24]. Compared with this report, the limit of detection for the ERA-based assay is approximately one order of magnitude more sensitive. Additional work would be required to incorporate reverse transcription for RNA detection, which could follow the optimization methods reported here.

To translate our methods into clinical use, point-of-care RNA or mRNA sample preparation methods need to be developed and integrated into the overall testing workflow. With the methods presented here, RNA extraction is required through a commercially available RNA extraction kit that costs approximately US$5 per extraction (based on the cost of New England Biolabs, Inc. Monarch RNA Extraction Kit, T2010). More importantly, the extraction process requires a centrifuge, a set of calibrated pipettes and pipette tips, and a skilled technician. Ongoing work includes incorporating point-of-care RNA extraction, which can build from promising research toward point-of-care nucleic acid extraction [25–30] and extraction-free RNA sample preparation [31]. Once sample preparation, amplification of additional HPV genotypes, and detection methods are integrated into a point-of-care test, larger-scale validation will be needed to verify clinical utility.

The work presented here demonstrates a low-cost method for HPV mRNA detection with clinically relevant analytical sensitivity and proof-of-concept compatibility with fluorimeters that are appropriate for use in resource-constrained settings. With the incorporation of integrated sample preparation and detection of additional genotypes, and with validation testing and further optimization to meet the WHO TPP criteria for point-of-care tests [22], this test has the potential to expand access to HPV mRNA testing and reduce overtreatment in resource-limited settings. As such, the methods presented in this report are a critical step toward achieving widespread HPV testing globally and provide a valuable blueprint for versatile DNA and/or RNA assay development for the additional targets that can be tailored based on infrastructure needs.

## Supplementary Information

Below is the link to the electronic supplementary material.

Supplementary file1 (DOCX 2322 KB)

## Declarations

### Source of biological material

Pre-existing and de-identified cervicovaginal swab samples were provided by the AIDS Malignancy Consortium (AMC).

### Copyright

Sections of this manuscript were previously published in KAK’s doctoral dissertation [32].

## References

[1] Singh D, Vignat J, Lorenzoni V, Eslahi M, Ginsburg O, Lauby-Secretan B, et al. Global estimates of incidence and mortality of cervical cancer in 2020: a baseline analysis of the WHO Global Cervical Cancer Elimination Initiative. Lancet Glob Health. 2023;11(2):e197–206. 36528031 10.1016/S2214-109X(22)00501-0 PMC9848409

[2] Cervical cancer [Internet]. World Health Organization. World Health Organization; [cited 2024 June 12]. Available from: https://www.who.int/news-room/fact-sheets/detail/cervical-cancer.

[3] World Health Organization (2014) Comprehensive cervical cancer control: a guide to essential practice, second edition. 25642554

[4] Sørbye SW, Fismen S, Gutteberg TJ, Mortensen ES, Skjeldestad FE. HPV mrna is more specific than HPV DNA in triage of women with minor cervical lesions. PLoS ONE. 2014;9(11):e112934. 25405981 10.1371/journal.pone.0112934 PMC4236101

[5] Sahasrabuddhe VV, Luhn P, Wentzensen N. Human papillomavirus and cervical cancer: biomarkers for improved prevention efforts. Future Microbiol. 2011;6(9):1083-98.6. 21958146 10.2217/fmb.11.87 PMC3809085

[6] Kelly H, Mayaud P, Segondy M, Pant Pai N, Peeling RW. A systematic review and meta-analysis of studies evaluating the performance of point-of-care tests for human papillomavirus screening. Sex Trans Infect. 2017;93(S4):S36-45. 10.1136/sextrans-2016-053070 29223961

[7] Iftner T, Becker S, Neis KJ, Castanon A, Iftner A, Holz B, et al. Head-to-head comparison of the RNA-based aptima human papillomavirus (HPV) assay and the DNA-based hybrid capture 2 HPV test in a routine screening population of women aged 30 to 60 years in Germany. J Clin Microbiol. 2015;53(8):2509–16. 26019212 10.1128/JCM.01013-15 PMC4508437

[8] Summary of commercially available HPV tests. [cited 2024b Jun 12]. Available from: https://www3.paho.org/hq/dmdocuments/2016/manual-VPH-English-02.pdf.

[9] Burd EM. Human papillomavirus laboratory testing: the changing paradigm. Clin Microbiol Rev. 2016;29(2):291–319. 26912568 10.1128/CMR.00013-15 PMC4786885

[10] Ratnam S, Coutlee F, Fontaine D, Bentley J, Escott N, Ghatage P, et al. Aptima HPV E6/E7 mRNA test is as sensitive as hybrid capture 2 assay but more specific at detecting cervical precancer and cancer. J Clin Microbiol. 2011;49(2):557–64. 21147950 10.1128/JCM.02147-10 PMC3043526

[11] MSF Access Campaign (2017) Putting HIV and HCV to the test: a product guide for point-of-care CD4 tests and laboratory-based and point-of-care HIV and HCV viral load tests, 3rd edition.

[12] Ting J, Smith JS, Myers ER. Cost-effectiveness of high-risk human papillomavirus testing with messenger RNA versus DNA under United States guidelines for cervical cancer screening. J Lower Gen Tract Dis. 2015;19(4):333–9. 10.1097/LGT.0000000000000143 26225945

[13] Kundrod KA, Barra M, Wilkinson A, Smith CA, Natoli ME, Chang MM, et al. An integrated isothermal nucleic acid amplification test to detect HPV16 and HPV18 DNA in resource-limited settings. Sci Transl Med. 2023;15(701):eabn4768. 37343083 10.1126/scitranslmed.abn4768 PMC10566637

[14] Van Doorslaer K, Li Z, Xirasagar S, et al. The papillomavirus episteme: a major update to the papillomavirus sequence database. Nucleic Acids Res. 2017. 10.1093/nar/gkw879. 28053164 10.1093/nar/gkw879 PMC5210616

[15] Van Doorslaer K, Tan Q, Xirasagar S, et al. The papillomavirus episteme: a central resource for papillomavirus sequence data and analysis. Nucleic Acids Res. 2013. 10.1093/nar/gks984. 23093593 10.1093/nar/gks984 PMC3531071

[16] Stier EA, Lensing SY, Darragh TM, Deshmukh AA, Einstein MH, Palefsky JM, et al. Prevalence of and risk factors for anal high-grade squamous intraepithelial lesions in women living with human immunodeficiency virus. Clin Infect Dis. 2019;70(8):1701–7. 10.1093/cid/ciz408 PMC7146000 31292602

[17] Coole J, Kortum A, Tang Y, Vohra I, Maker Y, Kundrod K, et al. Open-source miniature fluorimeter to monitor real-time isothermal nucleic acid amplification reactions in resource-limited settings. Journal of Visualized Experiments. 2021;(168). Available from: 10.3791/6214818. 10.3791/62148 33616108

[18] Natoli ME, Chang MM, Kundrod KA, Coole JB, et al. Allele-specific recombinase polymerase amplification to detect sickle cell disease in low-resource settings. Anal Chem. 2021;93(11):4832–40. 33689292 10.1021/acs.analchem.0c04191 PMC7992048

[19] Natoli ME, Kundrod KA, Chang MM, Smith CA, Paul S, Coole JB, et al. Improving performance of a SARS-CoV-2 RT-LAMP assay for use with a portable isothermal fluorimeter: towards a point-of-care molecular testing strategy. J Biomol Tech JBT. 2021;32(3):180–5. 35027875 10.7171/jbt.21-3203-013 PMC8730523

[20] Qian J, Boswell SA, Chidley C, Lu ZX, Pettit ME, Gaudio BL, et al. An enhanced isothermal amplification assay for viral detection. Nat Commun. 2020;11(1):5920. 33219228 10.1038/s41467-020-19258-y PMC7679446

[21] Derbie A, Mekonnen D, Woldeamanuel Y, et al. HPV E6/E7 mRNA test for the detection of high grade cervical intraepithelial neoplasia (CIN2+): a systematic review. Infect Agent Cancer. 2020;15:9. 32047531 10.1186/s13027-020-0278-x PMC7006188

[22] World Health Organization. Target product profiles for human papillomavirus screening tests to detect cervical pre-cancer and cancer. [cited 2024 Dec 27]. Available from: https://iris.who.int/bitstream/handle/10665/379099/9789240100275-eng.pdf.

[23] Lillis L, Siverson J, Lee A, Cantera J, Parker M, Piepenburg O, et al. Factors influencing Recombinase polymerase amplification (RPA) assay outcomes at point of care. Mol Cell Prob. 2016;30(2):74–8. 10.1016/j.mcp.2016.01.009 PMC4818709 26854117

[24] Ding N, Qi W, Wu Z, Zhang Y, Xu R, Lin Q, et al. Development of enzymatic recombinase amplification assays for the rapid visual detection of HPV16/18. J Microbiol Biotechnol. 2023;33(8):1091–100. 37635316 10.4014/jmb.2304.04009 PMC10468672

[25] Rodriguez NM, Linnes JC, Fan A, Ellenson CK, Pollock NR, Klapperich CM. Paper-based RNA extraction, in situ isothermal amplification, and lateral flow detection for low-cost, rapid diagnosis of influenza A (H1N1) from clinical specimens. Anal Chem. 2015;87(15):7872–9. 26125635 10.1021/acs.analchem.5b01594 PMC4878390

[26] Adams NM, Bordelon H, Wang KKA, Albert LE, Wright DW, Haselton FR. comparison of three magnetic bead surface functionalities for RNA extraction and detection. ACS Appl Mater Interf. 2015;7(11):6062–9. 10.1021/am506374t 25710198

[27] Bordelon H, Adams NM, Klemm AS, Russ PK, Williams JV, Talbot HK, et al. Development of a low-resource RNA extraction cassette based on surface tension valves. ACS Appl Mater Interf. 2011;3(6):2161–8. 10.1021/am2004009 PMC3129697 21604768

[28] Kolluri N, Albarran N, Fan A, Olson A, Sagar M, Young A, et al. SNAPflex: a paper-and-plastic device for instrument-free RNA and DNA extraction from whole blood. Lab on a Chip. 2020;20(18):3386–98. 32766666 10.1039/d0lc00277a PMC11556430

[29] Pearlman SI, Leelawong M, Richardson KA, Adams NM, Russ PK, Pask ME, et al. Low-resource nucleic acid extraction method enabled by high-gradient magnetic separation. ACS Appl Mater Interf. 2020;12(11):12457–67. 10.1021/acsami.9b21564 PMC7082792 32039572

[30] Horst AL, Rosenbohm JM, Kolluri N, Hardick J, Gaydos CA, Cabodi M, et al. A paperfluidic platform to detect Neisseria gonorrhoeae in clinical samples. Biomedical Microdevices. 2018;20(2):35. 29644437 10.1007/s10544-018-0280-x PMC6154386

[31] Delgado-Diaz DJ, Sakthivel D, Nguyen HHT, Farrokzhad K, Hopper W, Narh CA, et al. Strategies that facilitate extraction-free SARS-CoV-2 nucleic acid amplification tests. Viruses. 2022;14(6):1311. 35746782 10.3390/v14061311 PMC9230587

[32] Kundrod KA. Point-of-care tests to amplify and detect high-risk HPV DNA and mRNA [dissertation]. Houston, Texas (USA): Rice University; 2020.

[33] Higgins M, Ravenhall M, Ward D, Phelan J, Ibrahim A, Forrest MS, et al. PrimedRPA: primer design for recombinase polymerase amplification assays. Bioinformatics. 2018;35(4):682–4. 10.1093/bioinformatics/bty701 PMC6379019 30101342

[34] Da Conceicao Gomes Leitao M, Coimbra EC, Lima R de CP de, Guimarães M de L, Heráclio S de A, Silva Neto J da C, et al. Quantifying mRNA and microRNA with qPCR in cervical carcinogenesis: a validation of reference genes to ensure Accurate data. PLoS ONE. 2014;9(11):e111021. 10.1371/journal.pone.0111021 PMC4217744 25365304

[35] Hologic Aptima HPV Assay. APTIMA HPV assay. [cited 2024b Aug 29]. Available from: https://www.hologic.com/sites/default/files/package-insert/AW-14517-001_003_01.pdf.

[36] QuantiVirusTM HPV E6/E7 mRNA Test - DiaCarta, Inc.

[37] UNITAID. Screening and treatment of pre-cancerous lesions for ... [cited 2024b Aug 29]. Available from: https://unitaid.org/assets/Cervical_Cancer_Technology-landscape-2019.pdf.

[38] Molden T, Kraus I, Skomedal H, Nordstrøm T, Karlsen F. PreTectTM HPV-Proofer: Real-time detection and typing of E6/E7 mRNA from carcinogenic human papillomaviruses. J Virol Methods. 2007;142(1–2):204–12. 17379322 10.1016/j.jviromet.2007.01.036

[39] Spence RP, Murray A, Banks L, Kelland LR, Crawford L. Analysis of human papillomavirus sequences in cell lines recently derived from cervical cancers. Cancer Res. 1988;48(2):324–8. 2825972

