# Supplementary material for: A novel method for semi-quantitative detection of HPV16 and HPV18 mRNA with a low-cost, open-source fluorimeter
Source: Anal Bioanal Chem. 2025 Feb 7;417(9):1765–78. doi: 10.1007/s00216-025-05765-8 (PMC11913951; doi:10.1007/s00216-025-05765-8)
Supplement: Supplementary file 1 — Supplementary file1 (DOCX 2322 KB) [file 216_2025_5765_MOESM1_ESM.docx]

**Electronic Supplementary Material**

**Table S1: Assay compositions**

*Note:* *within each section, key differences in methods relative to the previous assay composition are shown in bold*

| **Fig.** | **SPX/ MPX** | **Target** | **Instru- ment** | **Rxn vol. (µL)** | **HPV16 reaction** | **HPV18 reaction** | **Combined HPV16/ HPV18 reaction** |
| --- | --- | --- | --- | --- | --- | --- | --- |
| **Section 1: exo assay optimization** | | | | | | | |
| S1 A | SPX | 10 µL gBlock DNA | T8-ISO or T16-ISO | 50 | RHB: 29.5 µL  16rF: 2.1 µL  16rP: 0.6 µL  16rR: 2.1 µL  Tte UvrD: 0-0.6 µL  MgOAc: 2.5 µL  H_2_O: 2.6-3.2 µL | N/A | N/A |
| S1 B | SPX | 10 µL gBlock DNA | T8-ISO or T16-ISO | 50 | RHB: 29.5 µL  16rF: 2.1 µL  16rP: 0.3-0.6 µL  16rR: 2.1 µL  Tte UvrD: 0.2 µL  MgOAc: 2.5 µL  H_2_O: 3.0-3.3 µL | N/A | N/A |
| S2 | SPX | 10 µL *in vitro* transcribed RNA | T8-ISO or T16-ISO | 50 | RHB: 29.5 µL  16rF: 2.1 µL  16rP: 0.3 µL  16rR: 2.1 µL  Tte UvrD: 0.2 µL  RNaseH: 1 µL  **dNTPs: 0-1.5 µL**  **RTase: 0-0.5 µL**  MgOAc: 2.5 µL  **H_2_O: to 50 µL** | RHB: 29.5 µL  18rF: 2.1 µL  18rP: 0.6 µL  18rR: 2.1 µL  RNaseH: 1 µL  **dNTPs: 0-1.5 µL**  **RTase: 0-0.5 µL**  MgOAc: 2.5 µL  **H_2_O: to 50 µL** | N/A |
| S3 | MPX | **10 µL gBlock DNA** | T8-ISO or T16-ISO | 50 | N/A | N/A | RHB: 29.5 µL  **16rF: 0.53-1.05 µL**  **16rP: 0.15-0.3 µL**  **16rR: 0.53-1.05 µL**  **18rF: 0.53-1.05 µL**  **18rP: 0.3-0.3 µL**  **18rR: 0.53-1.05 µL**  Tte UvrD: 0.2 µL  MgOAc: 2.5 µL  **H_2_O: to 50 µL** |
| **Section 2: Increasing sample complexity from synthetic DNA to extracted RNA from cervicovaginal swabs** | | | | | | | |
| 1, 2 | SPX | Fig 1: 10 µL gBlock DNA  Fig 2: 10 µL extracted cellular DNA | T8-ISO or T16-ISO | 50 | RHB: 29.5 µL  16rF: 2.1 µL  16rP: 0.3 µL  16rR: 2.1 µL  Tte UvrD: 0.2 µL  MgOAc: 2.5 µL  H_2_O: 3.3 µL | RHB: 29.5 µL  18rF: 2.1 µL  18rP: 0.6 µL  18rR: 2.1 µL  MgOAc: 2.5 µL  H_2_O: 3.2 µL | N/A |
| 3, 4 | SPX | Fig 3**: 10 µL** ***In vitro* transcribed RNA**  Fig 4: **10 µL extracted cellular RNA** | T8-ISO or T16-ISO | 50 | RHB: 29.5 µL  16rF: 2.1 µL  16rP: 0.3 µL  16rR: 2.1 µL  Tte UvrD: 0.2 µL  **RNaseH: 1 µL**  **dNTPs: 1.5 µL**  **RTase: 0.5 µL**  MgOAc: 2.5 µL  H_2_O: 0.3 µL | RHB: 29.5 µL  18rF: 2.1 µL  18rP: 0.6 µL  18rR: 2.1 µL  **RNaseH: 1 µL**  **dNTPs: 1.5 µL**  **RTase: 0.5 µL**  MgOAc: 2.5 µL  H_2_O: 0.2 µL | N/A |
| 5 | SPX | **10 µL extracted RNA from cervico-vaginal swabs** | T8-ISO or T16-ISO | 50 | RHB: 29.5 µL  16rF: 2.1 µL  16rP: 0.3 µL  16rR: 2.1 µL  Tte UvrD: 0.2 µL  RNaseH: 1 µL  dNTPs: 1.5 µL  RTase: 0.5 µL  MgOAc: 2.5 µL  H_2_O: 0.3 µL  (note: HPV16 data not shown) | RHB: 29.5 µL  18rF: 2.1 µL  18rP: 0.6 µL  18rR: 2.1 µL  RNaseH: 1 µL  dNTPs: 1.5 µL  RTase: 0.5 µL  MgOAc: 2.5 µL  H_2_O: 0.2 µL | N/A |
| **Section 3: Adapting assay to more point-of-care-friendly formats** | | | | | | | |
| 6 | **MPX** | **10 µL gBlock DNA** | T8-ISO or T16-ISO | 50 | N/A | N/A | **RHB: 29.5 µL**  **16rF: 0.79 µL**  **16rP: 0.15 µL**  **16rR: 0.79 µL**  **18rF: 0.79 µL**  **18rP: 0.3 µL**  **18rR: 0.79 µL**  **Tte UvrD: 0.2 µL**  **MgOAc: 2.5 µL**  **H_2_O: 4.2 µL** |
| 7A, 7B | **SPX** | **Variable volume of gBlock DNA (20% of total reaction volume)** | **Bio-Rad CFX96** | **Variable (5-50 µL)** | **Master mix prepared with following ratios, then aliquoted to 80% of total reaction volume**  **RHB: 29.5 µL**  **16rF: 2.1 µL**  **16rP: 0.3 µL**  **16rR: 2.1 µL**  **Tte UvrD: 0.2 µL**  **MgOAc: 2.5 µL**  **H_2_O: 3.3 µL** | **Master mix prepared with following ratios, then aliquoted to 80% of total reaction volume**  **RHB: 29.5 µL**  **18rF: 2.1 µL**  **18rP: 0.6 µL**  **18rR: 2.1 µL**  **MgOAc: 2.5 µL**  **H_2_O: 3.2 µL** | N/A |
| 7C, 7D | SPX | Variable volume of gBlock DNA (20% of total reaction volume) | **T8-ISO or T16-ISO** | Variable (5-50 µL) | Master mix prepared with following ratios, then aliquoted to 80% of total reaction volume  RHB: 29.5 µL  16rF: 2.1 µL  16rP: 0.3 µL  16rR: 2.1 µL  Tte UvrD: 0.2 µL  MgOAc: 2.5 µL  H_2_O: 3.3 µL | Master mix prepared with following ratios, then aliquoted to 80% of total reaction volume  RHB: 29.5 µL  18rF: 2.1 µL  18rP: 0.6 µL  18rR: 2.1 µL  MgOAc: 2.5 µL  H_2_O: 3.2 µL | N/A |
| 7E | SPX | **5 µL *in vitro* transcribed RNA** | **Low-cost, open-source fluorimeter** | **25 µL** | Master mix prepared with following ratios, then aliquoted to 20 µL  RHB: 29.5 µL  16rF: 2.1 µL  16rP: 0.3 µL  16rR: 2.1 µL  Tte UvrD: 0.2 µL  **RNaseH: 1 µL**  **dNTPs: 1.5 µL**  **RTase: 0.5 µL**  MgOAc: 2.5 µL  H_2_O: 0.3 µL | Master mix prepared with following ratios, then aliquoted to 20 µL  RHB: 29.5 µL  18rF: 2.1 µL  18rP: 0.6 µL  18rR: 2.1 µL  **RNaseH: 1 µL**  **dNTPs: 1.5 µL**  **RTase: 0.5 µL**  MgOAc: 2.5 µL  H_2_O: 0.2 µL | N/A |

*RHB: rehydration buffer; Tte UvrD: 20 µg/mL; RNaseH: 5,000 U/µL, dNTPs: 10 mM, RTase: 200 U/µL SuperScript IV Reverse Transcriptase; MgOAc: 280 mM magnesium acetate; H_2_O: nuclease-free water*

**Table S2: Primer and probe sequences**

| **Target** | **Assay** | **Type** | **Name** | **Sequence (5’ 🡪 3’)** | **Source** |
| --- | --- | --- | --- | --- | --- |
| HPV16 | PCR | Forward primer | 16qF | atgacagctcagaggaggagga | P3 |
|  | PCR | Reverse primer | 16qR | acaaccgaagcgtagagtcaca | P3 |
|  | RPA exo | Forward primer | 16rF | catggagatacacctacattgcatgaatat | pRPA |
|  | RPA exo | Probe | 16rP | caattaaatgacagctcagaggaggagga/i-fam/g/i-dsp/aa/i-tbhq1/agatggtccagctgg/3spc3 | pRPA |
|  | RPA exo | Reverse primer | 16rR | cacaaccgaagcgtagagtcacacttgcaa | pRPA |
| HPV18 | PCR | Forward primer | HPV18qF | ccgaaccacaacgtcacacaat | P3 |
|  | PCR | Reverse primer | HPV18qR | aaggtcgtctgctgagctttct | P3 |
|  | RPA exo | Forward primer | HPV18rF | aagacattgtattgcatttagagccccaaa | pRPA |
|  | RPA exo | Probe | HPV18rPM | cagaggaagaaaacgatgaaatagatggag/i-hex/t/i-dsp/a/i-tbhq1/catcaacatttacca/cspc3 | pRPA |
|  | RPA exo | Reverse primer | HPV18rRM | tactagctcaattctggcttcacacttaca | pRPA |
| Beta Actin | PCR | Forward primer | ACTBqF | tcgagcaagagatggccac | Leitão et al. |
|  | PCR | Reverse primer | ACTBqR | ggaaggaaggctggaagagt | Leitão et al. |

*Probe modifications are listed per Biosearch Technologies, Inc. conventions. PCR primers apply both to qPCR and RT-qPCR assays. RPA exo primers and probes apply both to RPA exo and RT-RPA exo assays. P3: Primer3 software; pRPA: PrimedRPA software* [33]*; modifications present between slashes—i-fam: internal fluorescein dT; i-dsp: internal 1’,2’ dideoxyribose (dSpacer); i-tbhq1: internal Black Hole Quencher®-1 dye; i-hex: internal HEX dT; 3spc3: 3’ C3 spacer. All primer sequences – and probes with adapted modifications – previously reported in* [13]*; beta actin primers published by Leitão et al.* [34].

**Fig. S1: Suppression of false positives arising from spurious primer-probe interactions in the HPV16 assay.** Seven no-target controls (NTCs) and one positive control of 10,000 input copies of HPV16 DNA were tested in each condition. (**A**) A range of volumes of Tte UvrD helicase (20 µg/mL) between 0 and 0.6 µL were added to each 50 µL reaction. The optimal condition was 0.2 µL per 50 µL reaction for an effective helicase concentration of 4 ng/reaction. (**B**) Probe concentrations of 150 nM and 300 nM were tested with 0.2 µL Tte UvrD helicase included in each 50 µL reaction. Reducing the amount of probe from manufacturer-recommended concentration of 300 nM to 150 nM reduced the magnitude of both false positives and true positives. With 150 nM probe and 4 ng helicase/50 µL reaction, false positives are suppressed, and true positives are maintained.

**Fig. S2: dNTP optimization for RNA detection.** Additional dNTPs ranging from 0 to 3mM were added to 50µL HPV18 RNA reactions with (+) or without (-) reverse transcriptase (RT). Ten thousand *in vitro* HPV18 transcripts were added to each reaction. The addition of 3mM dNTPs improved amplification efficiency without introducing false positives. *RFU: relative fluorescence units.*

**Fig. S3: Multiplex assay optimization.** A range of HPV16 and HPV18 primers and probes were tested to find a multiplex assay formulation that does not produce false positives and minimizes time-to-threshold for both targets. Each assay formulation (A-G) was tested with 6 no-target controls (NTCs), an HPV16 positive control, and an HPV18 positive control. Both positive controls were prepared at a concentration of 10,000 copies of HPV DNA per reaction. Standard multiplexed concentrations (condition A) produced false positives in 6/6 NTCs. Lowering the HPV16 probe to 30 nM markedly reduced false positives in conditions B-G. Condition F was determined to be the optimal formulation for false positive suppression (0/6 false positives) and time-to-threshold (7.8-8.8 minutes).

**Table S3: Reagent costs for a range of reaction volumes in singleplex and multiplex format**


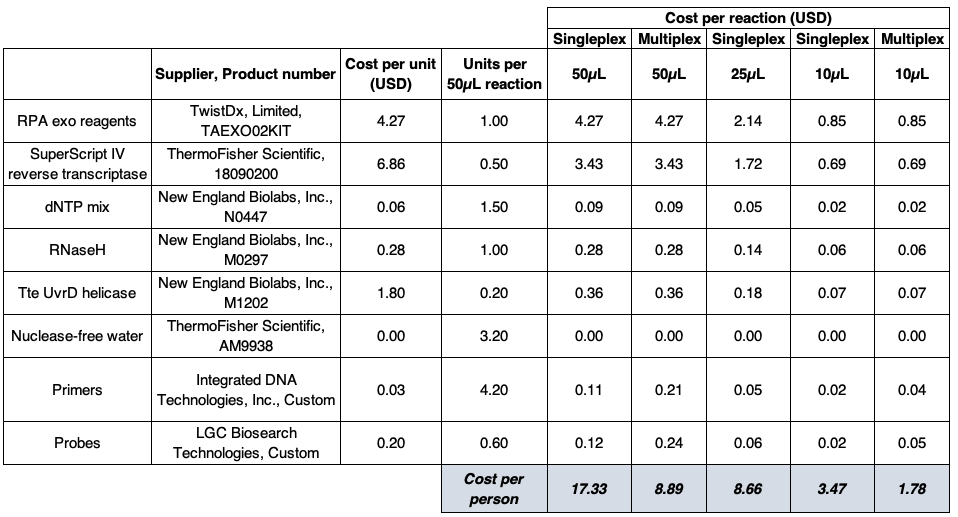


If a singleplex (SPX) assay is run, two reactions would be run per person—one for HPV16 and one for HPV18, thereby doubling the cost of reagents. If a multiplex (MPX) assay is run, a single assay would be run per person. The presented assay is characterized most thoroughly in singleplex 50 µL reaction format. In this report, results are shown for multiplex 50 µL reactions, singleplex 5-50µL DNA reactions, and singleplex 25 µL RNA reactions. We previously demonstrated the ability to multiplex 10 µL reactions relying on the same primers employed in this study with the detection of DNA by endpoint lateral flow instead of real-time fluorescence [13]. Moving forward, we aim to achieve RNA amplification and fluorescence detection in a multiplexed 10 µL reaction format, reducing the reagent cost per person to $1.78.

**Table S4: Summary of analytic sensitivity by target type**

| **Target** | **HPV16 limit of detection (copies/reaction)** | **HPV18 limit of detection (copies/reaction)** |
| --- | --- | --- |
| **Synthetic DNA** | 50 | 10 |
| **Extracted cellular DNA** | 1,000 | 1,000 |
| ***in vitro* transcribed RNA** | 1,000 | 100 |
| **Extracted cellular RNA** | 1,000 | 1,000 |

**Table S5: Summary of commercially available HPV mRNA tests**

| **Test** | **Genotypes detected** | **Commercially available?** | **Limit of detection** | **Clinical sensitivity range (%)** | **Clinical specificity range (%)** |
| --- | --- | --- | --- | --- | --- |
| **Aptima HPV (Hologic)** | All high-risk HPV | Yes | 44-50 *in vitro* transcripts/reaction [35] | 78.1-96.3 [21] | 25-96.1 [21] |
| **QuantiVirus HPV E6/E7 (DiaCarta)** | All high-risk HPV | Yes | 1,960 HPV18 copies within HeLa cell lysate [36] | 71.9-93.4 [21] | 15.5-85 [21] |
| **NucliSens EasyQ HPV (bioMérieux) / PreTect HPV-Proofer (PreTect AS)** | 16, HPV18, 31, 33, 45 | Discontinued [37] | 10 SiHa or CaSki cells; 1 HeLa cell per reaction [38]; estimated 10-6,000 HPV16 transcripts or 500 HPV18 transcripts [39] | 72-95 [21] | 45-92.5 [21] |
